# Identification of a Functionally Distinct Truncated BDNF mRNA Splice Variant and Protein in *Trachemys scripta elegans*

**DOI:** 10.1371/journal.pone.0067141

**Published:** 2013-06-25

**Authors:** Ganesh Ambigapathy, Zhaoqing Zheng, Wei Li, Joyce Keifer

**Affiliations:** Neuroscience Group, Division of Basic Biomedical Sciences, University of South Dakota Sanford School of Medicine, Vermillion, South Dakota, United States of America; Karlsruhe Institute of Technology, Germany

## Abstract

Brain-derived neurotrophic factor (BDNF) has a diverse functional role and complex pattern of gene expression. Alternative splicing of mRNA transcripts leads to further diversity of mRNAs and protein isoforms. Here, we describe the regulation of *BDNF* mRNA transcripts in an *in vitro* model of eyeblink classical conditioning and a unique transcript that forms a functionally distinct truncated BDNF protein isoform. Nine different mRNA transcripts from the *BDNF* gene of the pond turtle *Trachemys scripta elegans* (*tBDNF*) are selectively regulated during classical conditioning: exon I mRNA transcripts show no change, exon II transcripts are downregulated, while exon III transcripts are upregulated. One unique transcript that codes from exon II, *tBDNF2a*, contains a 40 base pair deletion in the protein coding exon that generates a truncated tBDNF protein. The truncated transcript and protein are expressed in the naïve untrained state and are fully repressed during conditioning when full-length mature tBDNF is expressed, thereby having an alternate pattern of expression in conditioning. Truncated BDNF is not restricted to turtles as a truncated mRNA splice variant has been described for the human *BDNF* gene. Further studies are required to determine the ubiquity of truncated BDNF alternative splice variants across species and the mechanisms of regulation and function of this newly recognized BDNF protein.

## Introduction

Brain-derived neurotrophic factor (BDNF) is critically involved in the signaling cascades that underlie neuronal growth, synapse formation, and synaptic modifications that accompany plasticity states such as long-term potentiation (LTP) and various forms of learning [1], [2]. The diverse functional role of BDNF and its intricate pattern of regulation are reflected in the complexity of the *BDNF* gene. Multiple promoters regulate the spatial and temporal expression of alternatively spliced *BDNF* mRNA transcripts that have unique 5′ non-coding exons linked to a common coding exon that generates the preproBDNF protein [2]–[7]. In mammals, each *BDNF* transcript also has a 3′ untranslated region (UTR) characterized as long or short depending on the position of two alternative polyadenylation sites. Evidence suggests that the 3′ UTRs function to regulate translation [8] and localized intracellular trafficking of *BDNF* mRNAs [9].

It is generally assumed that each *BDNF* transcript generates the same BDNF protein. Distinct mRNA transcripts are formed due to alternative splicing of the protein coding exon with different non-coding 5′ exons. The *BDNF* protein coding region is generally thought to undergo no alternative splicing. However, Liu et al. [4] reported that the human *BDNF* gene undergoes in-frame splicing in the coding region resulting in deletion of 144 base pairs (bp), referred to as transcript *BDNF7*, that was predicted to produce a truncated BDNF protein. Unfortunately, there were no functional studies to determine the expression pattern or potential role of such a truncated mature BDNF protein in that report.

To investigate the regulatory mechanisms controlling BDNF expression critical for the acquisition of learned responses, we studied an *in vitro* model of eyeblink classical conditioning from the turtle using an isolated brainstem preparation [10], [11]. The resistance of turtle brain tissue to hypoxia allows for extensive neural circuitry to be maintained in a dish for extended periods required for cellular/molecular studies of learning. Considerable experimental advantage is achieved since neuronal circuits are accessible to recording electrodes and application of pharmacological compounds or other molecules such as small interfering RNAs (siRNAs). In place of using a tone or airpuff as for behaving animals, stimulation of the auditory nerve (the “tone” conditioned stimulus, CS) is paired with the trigeminal nerve (the “airpuff” unconditioned stimulus, US) that results in acquisition of burst discharge in the abducens nerve representative of a neural analog of a blink conditioned response (CR). Acquisition of CRs is dependent on synaptic insertion of AMPA receptors (AMPARs), and BDNF is a key component in these signal transduction events [12]–[14]. Here we describe the conditioning-related up- or downregulation of *tBDNF* mRNA transcripts. One unique transcript undergoes a splicing event that deletes 40 bp from the protein coding exon that places the downstream mRNA out-of-frame forming a functionally distinct BDNF protein. Truncated tBDNF has an alternate pattern of expression compared to full-length mature tBDNF during classical conditioning. It is expressed in the naïve untrained state but is suppressed during conditioning when full-length tBDNF is expressed. The function and mechanisms of regulation of this newly discovered truncated BDNF protein has yet to be assessed. It will be essential to determine the ubiquity of truncated BDNF isoforms among species and to characterize their role in neuronal signaling and behavior.

## Materials and Methods

### Ethics Statement

Freshwater pond turtles, *Trachemys scripta elegans*, purchased from commercial suppliers were anesthetized by hypothermia until torpid and decapitated. All experiments involving the use of animals were performed in accordance with the recommendations in the Guide for the Care and Use of Laboratory Animals of the National Institutes of Health. The protocol was approved by the University of South Dakota Institutional Animal Care and Use Committee (protocol number: 92-08-11-14B).

### Training Procedures

Brainstems were transected at the levels of the trochlear and glossopharyngeal nerves and the cerebellum was removed as described previously [11]. The preparation was continuously bathed (2–4 ml/min) with physiological saline containing (in mM): 100 NaCl, 6 KCl, 40 NaHCO_3_, 2.6 CaCl_2_, 1.6 MgCl_2_, and 20 glucose, which was oxygenated with 95% O_2_/5% CO_2_ and maintained at room temperature (22–24°C) at pH 7.6. The number of brainstem preparations used, and therefore the number of animals represented in the data, is reported in the Results. Suction electrodes were used for stimulation and recording of cranial nerves. The US was a twofold threshold single shock applied to the trigeminal nerve and the CS was a 100 Hz, 1 s train stimulus applied to the ipsilateral auditory nerve that was below threshold amplitude required to produce activity in the abducens nerve. Neural responses were recorded from the ipsilateral abducens nerve that innervates the extraocular muscles controlling movements of the eye, nictitating membrane, and eyelid. The CS–US interval was 20 ms, which was defined as the time between the CS offset and the onset of the US. The intertrial interval between the paired stimuli was 30 s. A pairing session was composed of 50 CS–US presentations that lasts 25 minutes in duration for one complete session (C1). In experiments in which there is a second pairing session (C2), it is preceded by a 30 min rest period during which no stimuli were delivered. Conditioned responses were defined as abducens nerve activity that occurred during the CS and exceeded an amplitude of twofold above the baseline recording level. Pseudoconditioning and extinction training consisted of the same number of CS and US stimuli that were explicitly unpaired using a CS–US interval randomly selected between 300 ms and 25 s.

### RNA Isolation and cDNA Synthesis

To obtain the *tBDNF* mRNA sequences, total RNA was isolated from the turtle brainstem using the RNeasy Mini kit (Qiagen, Valencia, CA) according to the recommended protocol. DNase treatment of total RNA was performed by using a RNase-free DNase kit (Qiagen). Total RNA (1 µg) was used for first-strand synthesis using oligo(dT) primers and the Superscript II First-Strand Synthesis system (Invitrogen, Carlsbad, CA), and PCR was performed with gene specific primers designed within the conserved region of human and chicken *BDNF* (Table S1 in File S1). The amplified PCR products were purified, cloned into a pGEM-T Easy Vector (Promega, Madison, WI) and sequenced by Sanger’s dideoxy method on an automated DNA sequencer (Applied Biosystems 3730xl, Iowa State Univ. Sequencing Facility, Ames, IA). The *tBDNF* mRNA sequences were identified by BLAST.

### RNA Ligase-Mediated Rapid Amplification of cDNA Ends (RLM-RACE) Analysis

To obtain the full-length *tBDNF* transcripts, 5′ and 3′ RLM-RACE was performed using the FirstChoice RLM-RACE kit (Ambion, Austin, TX). Briefly, for 5′ RACE, 5 µg of total RNA was treated with calf intestine alkaline phosphatase (CIP) at 37°C for 1 hr followed by extraction using the acid phenol:chloroform method. Samples were then treated with tobacco acid pyrophosphatase (TAP) at 37°C for 1 hr to decap the mRNA. Then a 5′ RACE adapter was ligated with decapped mRNA using T4 RNA ligase for 1 hr at 37°C. The adapter-ligated RNA was reverse transcribed with the random Decamers primer and M-MLV Reverse Transcriptase at 42°C for 1 hr. For 3′ RACE, 1 µg of total RNA was reverse transcribed using a 3′ RACE adapter and M-MLV Reverse Transcriptase at 42°C for 1 hr. cDNA (2 µl of each) was used as a template for nested PCR with nested *BDNF* specific and adapter-specific primer pairs (see Table S1 in File S1 for a list of *BDNF* primers). PCR was performed using thermostable DNA Polymerase (Ambion) according to the manufacturer’s protocol. The reaction conditions were: initial denaturation at 94°C for 3 min, 30 cycles at 94°C for 30 sec, 60°C for 30 sec and 72°C for 1 min, and final extension at one cycle at 72°C for 10 min (Gradient Thermal Cycler, Eppendorf, USA). The resulting PCR products were run on a 2.0% agarose gel, bands were excised and purified, cloned into pGEM-T Easy Vector (Promega) and sequenced.

### Analysis of tBDNF Transcript Splice Variant Expression and PCR

In order to examine expression of the *tBDNF* mRNA splice variants, PCR was performed for naïve, pseudoconditioned and conditioned preparations. Real-time PCR could not be used here for quantification because the transcript sequences are overlapping and the required PCR product would be too long for optimal amplification. Total RNA was extracted from brainstems and an equal concentration (2.0 µg/sample) was reverse transcribed using a 3′ RACE adapter and M-MLV Reverse Transcriptase at 42°C for 1 hr. PCR was performed with three different sets of primers to amplify *tBDNF* transcripts using the Accuprime *Pfx* polymerase system (Invitrogen). Gene specific primers (Table S2 in File S1) were designed to amplify and estimate the level of expression using the Primer 3 Software package (version 4.0). To analyze the expression of the *tBDNF* transcripts, PCR was performed in 2 steps. 1 µl cDNA was amplified in a total volume of 25 µl using the Accuprime *Pfx* polymerase system. The primary PCR was carried out by using exon specific outer primers and 3′ outer primers. Conditions for the PCR reaction were: initial denaturation at 94°C for 2 min, 25 cycles at 94°C for 30 sec, 62°C for 30 sec (exon 2), 60°C for 30 sec (exon 1 and exon 3), 68°C for 2 min and final extension at 68°C for 10 min. The secondary PCR was carried out by using 1 µl of each primary PCR product as a template with exon specific inner primers and 3′ inner primers. Conditions for the PCR reaction were: initial denaturation at 94°C for 2 min, 30 cycles at 94°C for 30 sec, 60°C for 30 sec, 68°C for 2 min and followed by final extension at 68°C for 10 min. Samples were confirmed to be free of DNA contamination by performing reactions without reverse transcriptase. 10 µl of each PCR products was electrophoresed onto 2.0% agarose gel and stained with ethidium broide (0.5 µg/ml). Images of the amplified products were acquired and the density of each band with background subtraction was measured using the InGenius Bio Imaging System (Syngene, Frederick, MD). The levels of each exon of mRNA expression were normalized according to the density of β-actin bands for each sample. The primers for b-actin were: Forward, 5′ AGGGAAATCGTGCGTGACAT 3′; Reverse, 5′ ATGCCACAGGATTCCATACC 3′. The number of PCR cycles was optimized to maintain the amplification process within the linear range. The amplicons were cloned in the pGEM-T easy vector system and sequenced to confirm their identity.

### Western Blot Analysis

Brainstems were frozen in liquid nitrogen immediately after the physiological experiments and stored at −80°C. Tissue was homogenized in lysis buffer (20 mM Tris, pH 8.0; 1 mM EDTA, 1% Nonidet P-40, 0.15 M NaCl, 10 mM Na_4_P_2_O_7_, and 5% glycine) with a protease (Roche, Mannheim, Germany) and phosphatase inhibitor cocktail (Sigma, St. Louis, MO), rotated at 4°C for 2 h, centrifuged at 14,000 g for 20 min at 4°C, and the supernatants aliquoted and stored at −80°C. Protein sample concentrates were solubilized in 2× SDS/β-mercaptoethanol and boiled for 5 min before separation by 15% SDS-PAGE. After electrophoresis, membranes were blocked with 5% nonfat dry milk in Tris-buffered saline/0.1% Tween-20 for 1 h at room temperature. Membranes were incubated with primary antibody overnight at 4°C, washed, and incubated with horseradish peroxidase-conjugated secondary antibodies (1∶10,000) for 1 h at room temperature. The primary antibody was a polyclonal raised against amino acids 130–247 of human BDNF (#20981; Santa Cruz, CA). Proteins were detected by the ECL Plus chemiluminescence system (Amersham, Piscataway, NJ). Optical densities of the bands were determined relative to background levels using ImageJ.

### Two-Dimensional (2-D) Gel Electrophoresis and Protein Identification By Mass Spectrometry

Brainstem tissue was homogenized in lysis buffer (9.5 M urea, 4% CHAPS, 50 mM DTT, 0.4% Bio-Lyte ampholytes (pH 3–10), 1% protease inhibitor cocktail). The tissue was sonicated for approximately 30 s on ice, rocked for 1 h at room temperature followed by centrifugation at 14,000 g at 15°C for 30 min. The supernatant was recovered and 150 µg/200 µl of protein extracts from each individual tissue sample was analyzed using immobilized 11 cm pH gradient (IPG) strips with a pH range of 3 to 10 on an Ettan IPGphor3 system (GE Healthcare, Sweden). Samples were loaded by in-gel passive rehydration overnight at 20°C. Isoelectric focusing was started at 250 V for 2 h, slowly increased to 8,000 V over 2.5 h, and held at 8000 V until 24,000 Vh was reached. Thereafter, the focused strips were equilibrated in equilibration buffer I (6 M urea, 2% SDS, 0.375 M Tris-HCl, pH 8.8, 20% glycerol, 130 mM DTT) for 15 min, followed by equilibration buffer II (6 M urea, 2% SDS, 0.375 M Tris-HCl, pH 8.8, 20% glycerol, 135 mM iodoacetamide) for 15 min. Afterwards, strips were placed on the top of 15% Tris-HCl SDS-polyacrylamide gels followed by electrophoresis. The truncated *tBDNF* splice variant was detected with a BDNF antibody from pseudoconditioned samples by western blot. For protein detection, SDS-polyacrylamide gels were fixed by 10% methanol and 7% acetic acid for 30 min, stained with Sypro Ruby (Bio-Rad Laboratories) overnight, destained in 10% methanol and 7% acetic acid for 1 h and rinsed. Protein spots were visualized using a Typhoon 9410 Workstation Fluorescence Scanner (Amersham-GE). Raw images of the 2-DE gels were imported into the 2-DE gel analysis software PDQuest 8.0 (Bio-Rad). The corresponding truncated tBDNF spot was excised with a spotcutter (Bio-Rad).

The excised spot was in-gel digested using sequencing grade trypsin (Promega) and nanoRPLC in-line desalted at 37°C overnight. The eluted ions were analyzed by one full precursor MS scan (400–1500 m/z) followed by four MS/MS scans of the most abundant ions detected in the precursor MS scan while operating under dynamic exclusion or a direct data acquisition system. Spectra obtained in the positive ion mode with a nano ESI-Q-Tof micro mass spectrometer (Micromass, Manchester, UK) were deconvoluted and analyzed using the MassLynx software 4.1 (Micromass). A peak list (PKL format) was generated to identify +1 or multiple charged precursor ions from the mass spectrometry data file. The instrument was calibrated in MS/MS mode using 500 fmole of human (Glu^1^)-Fibrinopeptide B with a RMS residual of 3.495 e-3 amu or 7.722 e^0^ ppm. Parent mass (MS) and fragment mass (MS/MS) peak ranges were 400–1500 Da and 65–1500 Da, respectively.

The Mascot Server (www.matrix-science.com) in MS/MS ion search mode and ProteinLynx Global Server (PLGS v2.4, Micromass) were applied to conduct peptide matches (peptide masses and sequence tags) and protein searches against NCBInr v20120825 (19981550 sequences; 6844480447 residues) using the taxonomy filter Eukaryota (eukaryotes) (6202072 sequences) and an individual database for BDNF (gi|295986644). The following parameters were set for the search: carbamidomethyl (C) on cysteine was set as fixed; variable modifications included asparagine and glutamine deamidation and methionine oxidation. Only one missed cleavage was allowed; monoisotopic masses were counted, the precursor peptide mass tolerance was set at 2 Da, fragment mass tolerance was 0.3 Da, and the ion score or expected cut-off was set at 5 Da. Known keratin contaminant ions were excluded. The MS/MS spectra were searched with MASCOT using a 95% confidence interval threshold (*P*<0.05) with which a minimum score of 58 was used for peptide identification.

### Statistical Analysis

Data were analyzed with StatView software using a one-way ANOVA followed by post hoc analysis using a Fisher’s test. Values are presented as means +/− SEM. *P* values are given in the text and are determined relative to the naïve group. Significant differences were considered to be *P*<0.05.

## Results

### Structure of the *tBDNF* Gene and Alternative Splicing of Transcripts

**Figure 1:**
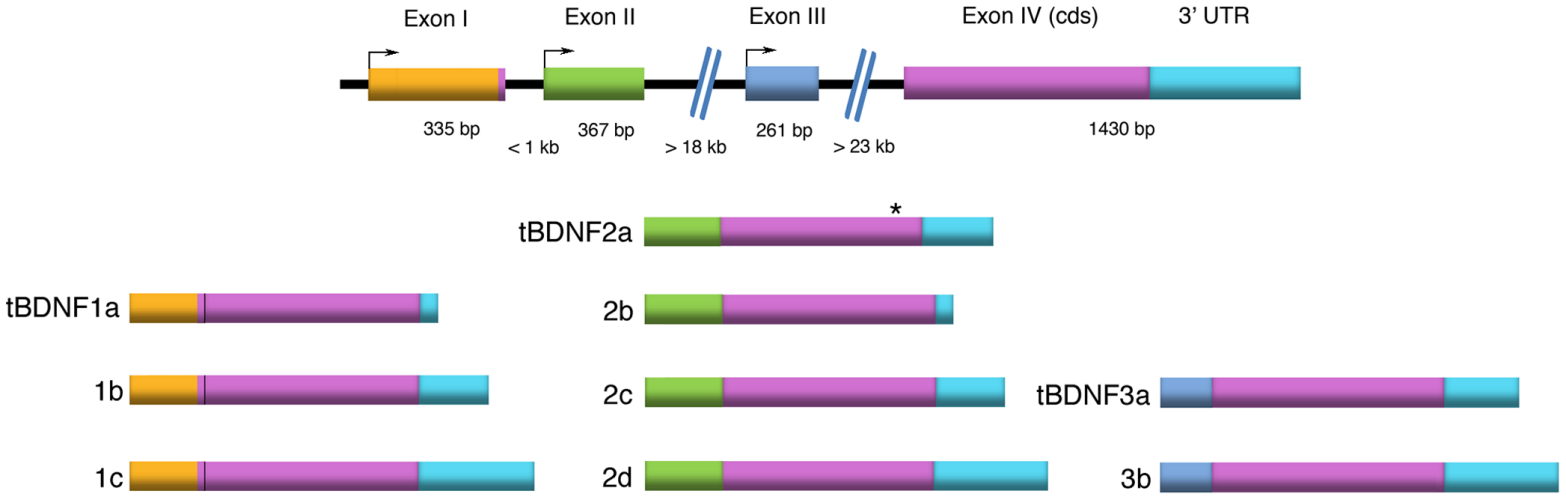
*BDNF* gene structure in turtle and alternatively spliced mRNA transcripts. Schematic illustration of the *BDNF* gene structure in turtle and the nine alternatively spliced mRNA transcripts identified here. The 5′ exons I, II, and III are spliced to a common protein coding exon IV that encodes the preproBDNF protein to form three classes of transcripts designated *tBDNF1-3*. Exon I contains a translation initiation site that codes for 8 additional amino acids at the N-terminal end of the BDNF protein and exons II and III are non-coding exons. Due to alternative splicing of three polyadenylation sites, the transcripts may have short, intermediate or long 3′ UTRs. One unique transcript, *tBDNF2a*, has a deletion within the coding exon (asterisk) that results in a truncated *tBDNF* mRNA transcript.

Since the complete *T. scripta elegans* chromosome sequence has not yet been identified, in order to obtain the mRNA sequence for *tBDNF* the human and chicken *BDNF* mRNA sequences were retrieved from the NCBI database and aligned using the MegAlign program DNASTAR. *BDNF* primers (Table S1 in File S1) were designed based on conserved regions for human and chicken *BDNF* to amplify the complete sequence of *tBDNF* and the resulting PCR products were cloned and sequenced. To analyze the *BDNF* gene structure and mRNA transcripts from the pond turtle *T. scripta elegans*, 3′ and 5′ RACE followed by PCR analysis was performed. Four different exons have been identified thus far and were named exon I, II, III and IV. Figure 1 shows a schematic diagram of the exon structure of the *tBDNF* gene. Exons I–III code for the 5′ flanking region and exon IV codes for the BDNF protein along with the 3′ UTR. Comparison by BLAST search shows that *tBDNF* exon I is 92% similar to human exon I, exons II and III are 72% similar to human exon II and IV, respectively, and the coding exon IV is 86% similar to the human coding exon IX. Additionally, as in the human, rodent [6] and chicken [15], exon I in turtle contains an ATG codon that is used as a translation initiation site leading to synthesis of a preproBDNF protein with an N-terminal sequence of 8 additional amino acids (Fig. 1). Exons II and III are untranslated exons.

**Figure 2:**
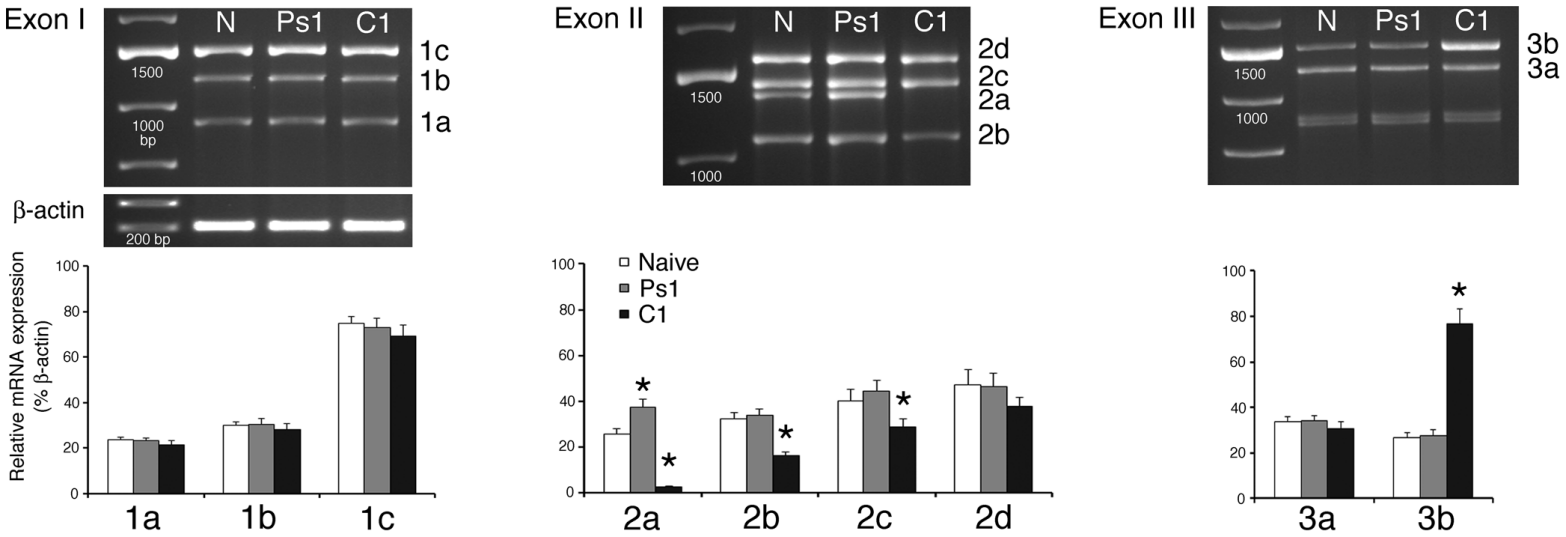
Expression of *tBDNF* mRNA transcripts is selectively regulated during conditioning. Agarose gels of the PCR products for each of the nine *tBDNF* mRNA transcripts showing their pattern of expression during the different training procedures examined: naïve (N), pseudoconditioned for one session (Ps1), and conditioned for one session (C1, or after 25 minutes). PCR products for b-actin mRNA transcripts were used for comparison and are shown. Semi-quantitative analysis of mRNA expression of each *tBDNF* transcript is shown. Transcripts *tBDNF2a-c* are significantly decreased after conditioning while *tBDNF3b* is increased by about threefold. The band below 1000 bp for the exon III gel is non-specific.

Nine different *tBDNF* transcripts designated *tBDNF1-3* after their 5′ exons are generated by alternative splicing (Fig. 1). Selective sets of primers were designed for PCR analysis (Table S2 and Fig. S1 in File S1) and identified the *tBDNF* mRNA splice variants for each of exons I, II, and III spliced to the coding exon IV and resulted in specific PCR products (Fig. 2). Each of the bands was sequenced to verify that they selectively represented each of the *tBDNF* transcripts. Naïve untrained brain tissue (N) shows PCR products for exon I at 943 bp, 1319 bp, and 1510 bp that correspond to mRNA transcripts *tBDNF1a-c*. Similarly, the primer set for exon II shows bands at 1089 bp, 1425 bp, 1465 bp, and 1656 bp corresponding to *tBDNF2a-d* mRNA transcripts. Transcript *tBDNF2a* (1425 bp) is unique in that it is similar to the other transcripts coded by exon II except that 40 bp are spliced out of the protein coding sequence and is only detectable on agarose gels when separated from transcript *tBDNF2c* that contains the full-length coding exon IV. Finally, a third primer set reveals bands for exon III at 1357 bp and 1548 bp corresponding to transcripts *tBDNF3a-b*. 3′ RACE analysis shows that all of the *tBDNF* transcripts *1-3* share the common coding exon IV but differ in their 3′ UTRs. Three alternative polyadenylation sites are present in exon IV which terminate each transcript resulting in *tBDNF* splice variants with short, intermediate, or long 3′ UTRs (Fig. 1). The complete cDNA sequences for the *tBDNF* transcripts are available in the GenBank database (accession numbers: KC151264– KC151272) and sequences showing isoform-specific primers are illustrated in Fig. S1 in File S1.

### Sequence Alignment Between Human and Turtle Full-Length and Truncated BDNF Proteins

**Figure 3:**
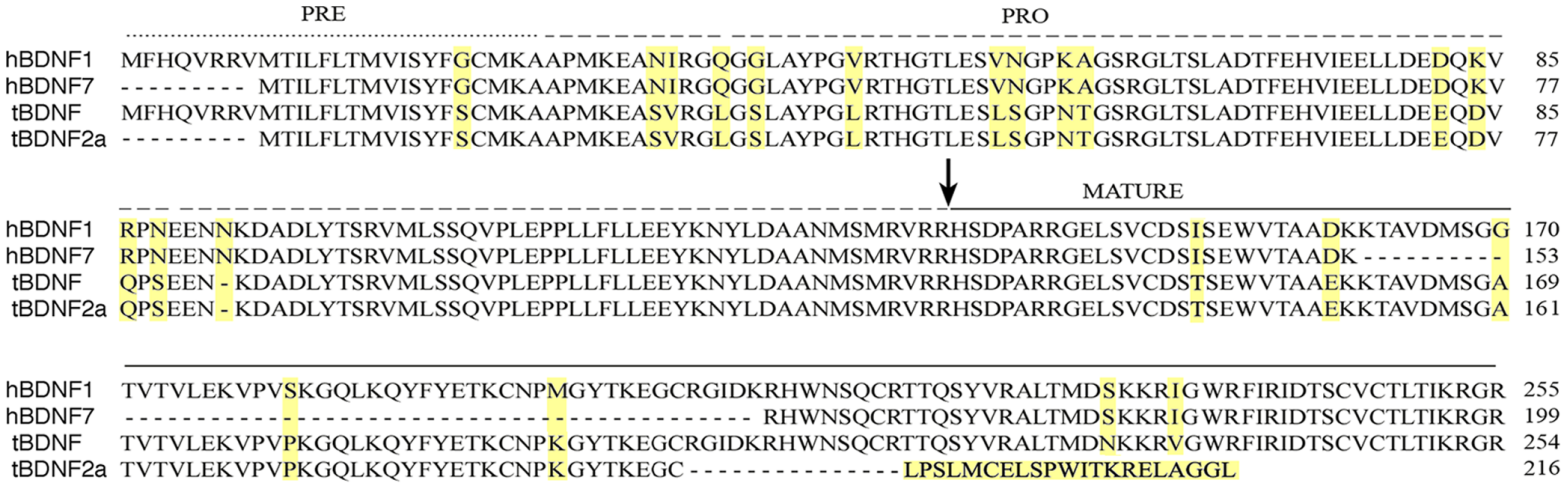
Comparison of the amino acid sequence between full-length and truncated human and turtle BDNF. Amino acid sequence alignment of the coding region for human BDNF 1 and 7 are shown aligned with turtle full-length BDNF and truncated BDNF (labeled as tBDNF2a) to show the two different truncated BDNF sequences. The position of the deleted amino acid sequences for truncated hBDNF7 and tBDNF2a are indicted by the dashes. Differences between human and turtle sequences are highlighted in yellow. The arrow indicates the site of proteolytic cleavage of mature BDNF protein from the proBDNF precursor.

A *BDNF* mRNA transcript that shows alternative splicing within the coding exon reported here for *tBDNF* has also been described previously in humans as *BDNF7* (GenBank accession number AY054406; ref. 4). Other mRNAs resulting from internal splicing of the human *BDNF* coding exon may also occur [7]. For comparison of the human and turtle truncated BDNF proteins, we performed an amino acid sequence alignment of the full-length and spliced preproBDNF proteins using the alignment program CLUSTAL W 2.0 (16; Fig. 3). Full-length human (h) BDNF1 has 255 amino acids in total (including 8 additional amino acids in the N-terminal sequence coded by exon I). An alternative in-frame splicing event in the *hBDNF7* coding exon deletes 144 bp in the region coding for mature BDNF that results in 48 amino acids fewer than the complete hBDNF1 mature protein [4]. Therefore, hBDNF7 has 199 amino acids and a predicted molecular weight of the mature protein of 8.2 kD. For comparison, tBDNF has 254 amino acids in total, however, *tBDNF2a* has 40 bp spliced out of the mature coding region that results in a deletion of 13 amino acids and a shift in the reading frame that produces a novel amino acid sequence at the C-terminal end. Due to this out-of-frame splicing event, *tBDNF2a* has a different stop codon compared with *tBDNF* that occurs earlier and generates a truncated protein of 216 amino acids. The predicted molecular weight of mature tBDNF from all of the alternatively spliced *tBDNF* mRNA variants is 13.6 kD except for truncated tBDNF which is 9.8 kD, and these values are subject to post-translational modifications that typically occur *in situ*. Therefore, due to alternative splicing events, both the human and turtle genomes code for a truncated mature BDNF protein although the truncations are markedly different from one another and result in proteins of unknown function.

### Expression of tBDNF mRNA Transcripts and Truncated Protein in Conditioning

**Figure 4:**
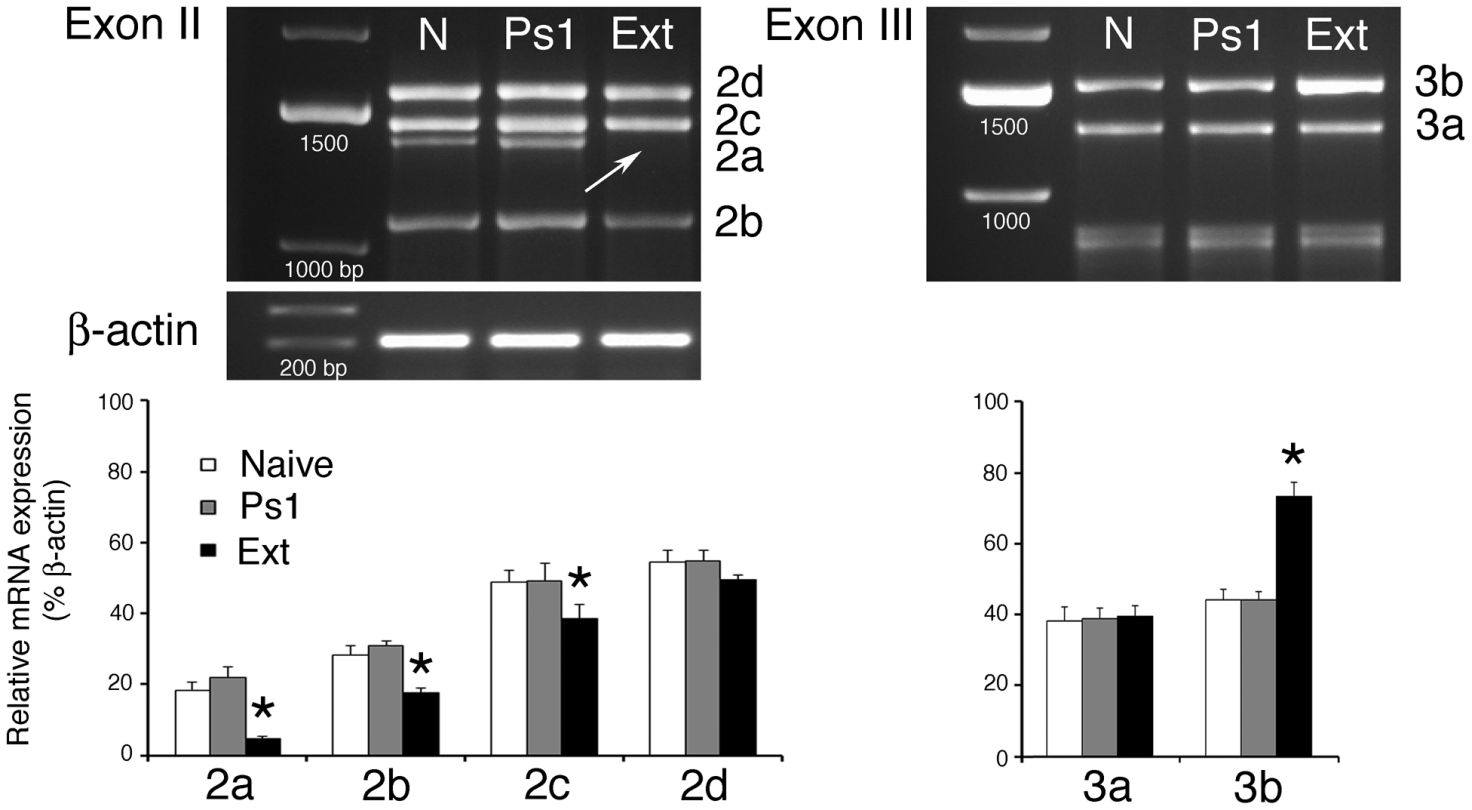
Expression of *tBDNF* mRNA transcripts after extinction training. Agarose gels showing PCR products for *tBDNF2* and *tBDNF3* mRNA transcripts during different training conditions including naïve (N), pseudoconditioning for one session (Ps1), and extinction training (Ext) using unpaired trials. After extinction training (in which preparations were conditioned to 100% CRs for two sessions and extinguished to 0% CRs after four or five sessions of unpaired stimuli) the pattern of transcript expression is remarkably similar to that observed after conditioning trials. Significantly, the 1425 bp band (arrow) representative of *tBDNF2a* is completely suppressed during extinction even though there are 0% CRs as recorded in the pseudoconditioned cases.

The expression pattern of full-length mature tBDNF protein and its function in *in vitro* classical conditioning has been well characterized in our model system [12], [13], [17]–[19]. ProBDNF protein isoforms are present under all conditions while full-length mature BDNF protein is expressed following proteolytic cleavage of the proBDNF precursor after one session of conditioning (or after 25 minutes of pairing; ref. 17) where it is required for synaptic AMPAR delivery and acquisition of CRs [12], [13]. In order to investigate the regulatory mechanisms controlling *tBDNF* expression during conditioning, we analyzed the level of *tBDNF1-3* transcripts in naïve, pseudoconditioned and conditioned preparations using PCR (using primer sets shown in Table S2 and Fig. S1 in File S1; Fig. 2, *n* = 5 brainstem preparations/group each analyzed for exons I, II and III). After one session of unpaired pseudoconditioning trials (Ps1; which is 25 minutes in duration) there are no significant changes in the levels of transcript expression compared to naïve except that truncated *tBDNF2a* is slightly increased (*P*<0.05). Following one session of conditioning (C1), the overall distribution of mRNA transcript expression across exons II–III is dramatically altered. The *tBDNF* splice variants having exon I are not regulated during conditioning while those with exon II are significantly downregulated, specifically *tBDNF2a*, *2b*, and *2c* (*P*<0.001 vs Naïve; Fig. 2). In fact, the truncated splice variant *tBDNF2a* was nearly completely suppressed after conditioning (*P*<0.0001 vs. Naïve) as shown in the gel where the band is not apparent (Fig. 2). In contrast, exon III transcript *tBDNF3b* is significantly upregulated with conditioning to threefold its naïve level (*P*<0.0001). Increased expression of this transcript after one session of conditioning may be responsible for the expression of mature BDNF protein at this time point, but this remains to be fully examined. We were next interested in examining whether truncated *tBDNF2a* mRNA was only expressed when the level of conditioning was low or zero, such as in the naïve state or during pseudoconditioning, and was re-expressed after extinction training. Extinction training is a procedure in which learned responses are initially acquired and then decline when they are no longer paired with a reinforcing stimulus. After extinction training in which CRs were acquired to 100% and then extinguished to 0% by several sessions of unpaired stimulation, PCR analysis showed that the band at 1425 bp identifying *tBDNF2a* was not expressed (Ext lane, arrow; Fig. 4, *P*<0.0001, *n* = 4 brainstem preparations/group). Therefore, we did not observe rapid re-expression of *tBDNF2a* transcription even though the level of CRs was 0% similar to the pseudoconditioned cases. Restoration of the *tBDNF2a* band failed to occur after 5 sessions of unpaired stimuli (or in about 4.5 hours) even though it was repressed after only one conditioning session (25 minutes). Further analysis of all the Exon II and III transcripts (Fig. 4) showed a pattern of expression remarkably similar to the conditioned state (Fig. 2) except that during extinction CRs were not expressed. While on the surface this finding seems perplexing, it is generally considered that extinction training is a form of new learning [20] which is consistent with the idea that repression of *tBDNF2a*, and the expression pattern of the other transcripts, is related to mechanisms underlying learning.

**Figure 5:**
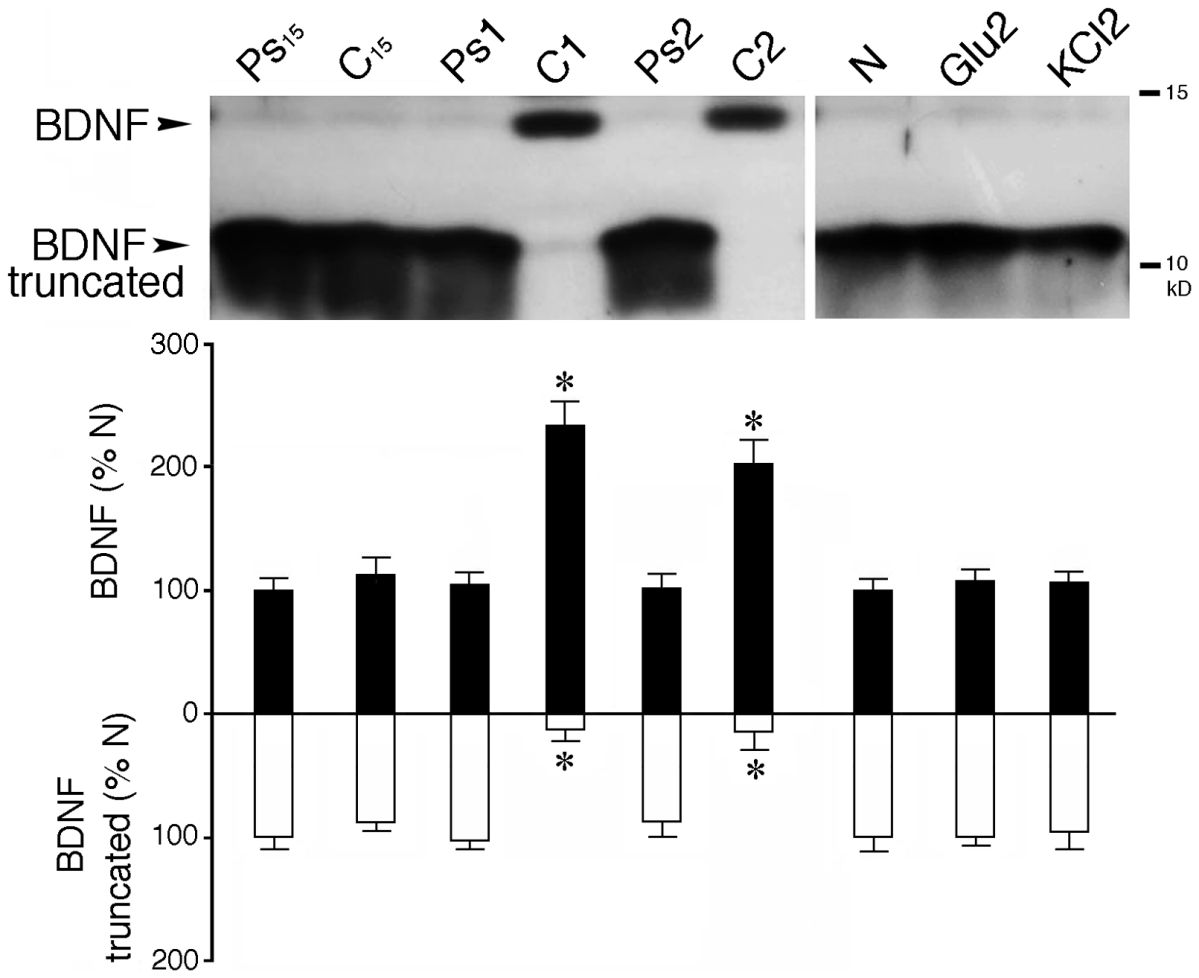
Conditioning induces expression of full-length mature tBDNF protein and suppression of truncated tBDNF. Western blot analysis using a primary antibody to BDNF showing expression of the full-length 14 kD mature tBDNF protein after one (C1) or two (C2) sessions of conditioning. In the naïve state (N), after pseudoconditioning trials for 15 minutes (Ps_15_) or one session (Ps1) or early stages of conditioning after 15 minutes (C_15_), an 11 kD protein band is expressed that represents a truncated tBDNF protein generated by the *tBDNF2a* transcript. When full-length mature tBDNF is expressed in conditioning, truncated tBDNF is completely suppressed. Expression of either protein isoform is not altered from the naïve state by a generalized increase in activity levels induced by bath application of 50 µM glutamate (Glu2) or 15 mM KCl (KCl2) for the equivalent time period of two sessions. *P* values are given in the text and are determined relative to the naïve group.

We next examined changes in tBDNF protein expression during conditioning. Our evidence indicates that the *tBDNF2a* transcript forms a functionally distinct ∼11 kD truncated BDNF protein that has an alternate pattern of expression compared to the ∼14 kD full-length mature BDNF during classical conditioning (Fig. 5, *n* = 5 brainstem preparations/group). Expression of mature tBDNF is activated after one session of conditioning (C1 or 25 minutes, *P*<0.0001) but not after 15 minutes (Fig. 5; C_15_, *P* = 0.67), as described previously [17]. In contrast, the 11 kD band is strongly expressed in naïve untrained preparations and during pseudoconditioning but is completely suppressed when full-length mature BDNF is expressed during conditioning for C1 or C2 (Fig. 5; *P*<0.0001). Moreover, this pattern of protein expression appears to be related specifically to the conditioning stimuli as increasing the general level of neuronal activity by bath application of 50 µM glutamate or 15 mM KCl for the equivalent time period of two pairing sessions has no effect on expression or repression of either protein isoform from the naïve state. Therefore, there is an alternate pattern of protein expression between the full-length mature tBDNF and the truncated protein during classical conditioning.

**Figure 6:**
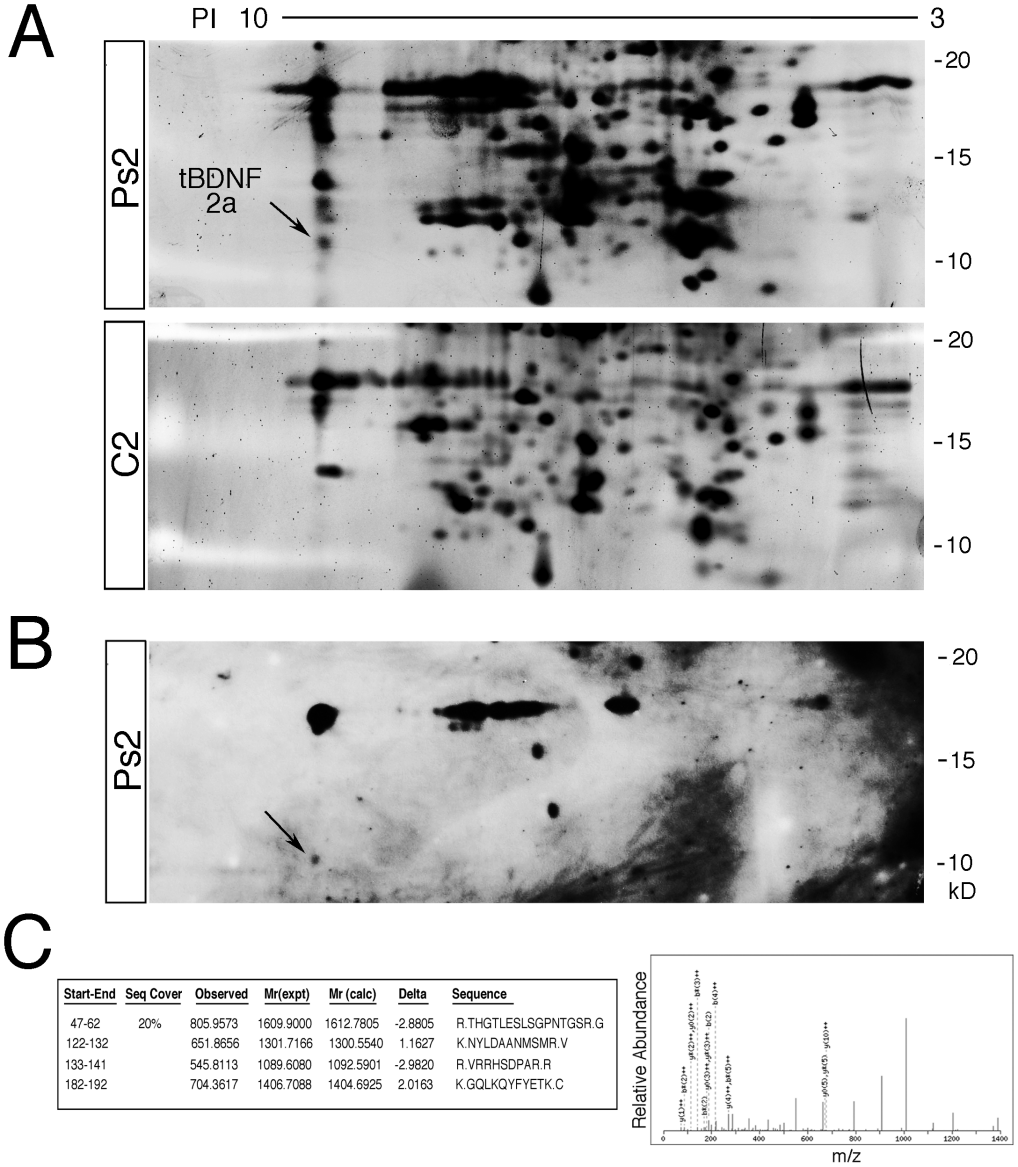
Identification of truncated tBDNF protein by 2-DE and mass spectrometry. (A) 2-D gels stained with Sypro-Ruby from a preparation that was pseudoconditioned for two sessions (Ps2) and one that was conditioned for two sessions (C2). The arrow (tBDNF2a) in the Ps2 gel indicates the spot that was excised for MS/MS analysis at ∼11 kD. (B) 2-D western blot from a pseudoconditioned preparation that was stained with a primary antibody to BDNF. The arrow indicates the ∼11 kD protein. (C) MS/MS analysis of the spot excised from 2-D gels prepared from pseudoconditioned preparations identified several peptides of BDNF in all of the samples tested. The quantitative data shown are from one sample in which four peptides were identified, showing the residue number, sequence coverage, masses, and sequences. The MS/MS spectrum is shown for tBDNF peptide NYLDAANMSMR from the sample shown in the table.

Finally, to confirm that the 11 kD protein observed in the western blots was indeed an isoform of the BDNF protein, we employed 2-DE and mass spectrometry for protein identification (Fig. 6). First, 2-D gels were obtained from pseudoconditioned and conditioned preparations and the position of the 11 kD protein spot of interest was identified in pseudoconditioned but not conditioned samples (Fig. 6*A*, *n* = 6 brainstem preparations/group that were examined by 2-DE). The position of the 11 kD protein was further confirmed by immunoblotting the same 2-D gels using a BDNF antibody (Fig. 6*B*). The 11 kD spot was excised and several peptide sequences were identified by tandem mass spectrometry (MS/MS) for each sample as matching to BDNF (Fig. 6*C*, *n* = 3 pseudoconditioned brainstem preparations that were analyzed by MS/MS). Data from one pseudoconditioned sample are summarized in the MS quantitative analysis (Fig. 6*C*). Four peptides corresponding to sequences of BDNF were identified and the MS/MS spectrum for the second peptide is shown. The identified peptides in the samples span a range throughout the BDNF sequence making it unlikely that the 11 kD spot on the 2-D gels was a degradation product. While the peptides identified in the samples were common to all of the *tBDNF* variants, only the truncated protein from *tBDNF2a* has a predicted molecular weight of 9.8 kD which closely matches the ∼11 kD band we observed in the western blots and the spot excised from the 2-D gels. Together, these results suggest that the truncated tBDNF protein is expressed during the resting or untrained state and is suppressed during the acquisition of learning when the full-length mature tBDNF isoform is expressed.

## Discussion

We present evidence for a newly recognized mRNA splice variant of *BDNF* that is translated into a functionally distinct truncated BDNF protein in turtles. The deletion of 40 bp in the protein coding exon by alternative splicing generates a *tBDNF2a* transcript that shifts the reading frame and produces an early stop codon that generates a truncated BDNF protein isoform lacking a total of 38 amino acids. The frame shift also results in a C-terminal amino acid sequence that is distinct from the prototypical mature tBDNF protein. While a number of alternatively spliced *BDNF* transcripts have been described in several mammalian [3]–[7] and non-mammalian [15] vertebrates that are produced by the splicing of unique non-coding 5′ exons to the protein coding sequence, it has generally been assumed that the coding region itself generates the same preproBDNF protein. However, this conclusion requires further scrutiny as suggested by our data from turtle and previous work obtained from human brain tissue [4]. Since BDNF is ubiquitous in brain and is a highly conserved gene it seems likely that alternative splicing within the protein coding exon may occur under some conditions in other species that results in a unique truncated BDNF protein isoform. Such mRNA alternative splicing events that generate truncated protein products with functions distinct from the full-length protein have recently been recognized, for example, for the tropomyosin-related kinase B (TrkB) receptor [21]. Possible reasons for why a truncated BDNF protein isoform may have gone unrecognized until now and the potential functions of this protein in learning are discussed below.

### Identification of a Truncated BDNF Protein Isoform

In our earlier studies of the role of BDNF in *in vitro* classical conditioning [12], [17] we observed an 11 kD protein in western blots of BDNF whose expression was turned off when the 14 kD mature BDNF protein was expressed during acquisition of CRs. We have discovered a *tBDNF* mRNA transcript, *tBDNF2a*, that has a deletion in the protein coding exon and have identified the 11 kD protein band as an isoform of BDNF using mass spectrometry. While a truncated *BDNF* mRNA transcript originating from the protein coding exon has also been reported for human [4], [7], it is unclear why such splice variants have not been reported for other species if they are indeed present and expressed in high enough amounts to be detected. This is particularly perplexing for studies on rodents that are commonly used in behavioral and cellular studies of activity-dependent and learning-induced BDNF expression. There are several possible explanations for why this might be the case for both the mRNA and protein. First, favorable behavioral conditions for expression of an alternatively spliced *BDNF* mRNA from the coding exon may not have been examined. In our study, we observed the highest levels of *tBDNF2a* mRNA and protein expression after unpaired pseudoconditioning trials. Second, there are technical considerations related to the mRNA detection. Primers used for PCR of *BDNF* may not be designed to detect short deletions if their location is unexpected or unknown. Additionally, as in the case of *tBDNF2a*, only 40 bp are deleted and due to this short sequence the PCR product cannot be separated from others in standard 1–1.5% agarose gels (2.0–3.2% gels were used here). Third, for protein visualization, the primary antibody used in our earlier studies (12; Santa Cruz, #20981) was raised against a sequence localized to mature human BDNF. While this antibody is still used to visualize the proBDNF and mature BDNF proteins, it no longer recognizes the 11 kD band in turtle and we are aware of no other antibodies that currently detect it for turtle or rodent brain tissue. It is intriguing that a smaller molecular weight band appears below mature BDNF in an early study using a different BDNF antibody in adult mouse brain [22; their Supplementary Fig. 1] suggesting the possibility of a truncated protein in that species. This highlights the need for further studies clarifying whether truncated *BDNF* mRNA and protein isoforms exist in measurable amounts in mammals and under what conditions.

### Regulation of Alternatively Spliced *tBDNF* mRNA and Protein Expression in Conditioning

The expression of alternatively spliced *tBDNF* mRNAs is clearly under transcriptional control during classical conditioning. The *tBDNF2a-c* mRNA transcripts are significantly downregulated during conditioning while *tBDNF3b* is upregulated. Strikingly, the truncated transcript *tBDNF2a* is completely repressed in conditioning. It is interesting that the *tBDNF3b* transcript, which is uniquely upregulated in conditioning, has a long 3′ UTR. Previous studies have found that the long 3′ UTR, but not the short one, allows for rapid activity-dependent translation of mature BDNF [8] and controls the subcellular targeting of *Bdnf* mRNAs [9]. These observations have important structure/function implications since *tBDNF* transcripts encoded by the same 5′ exon but with different 3′ UTRs undergo differential expression. *BDNF* gene transcription is regulated by alterations in epigenetic factors such as levels of DNA methylation and histone modification during synaptic plasticity and learning [23, 24; reviewed in ref. 2]. Methylated CpG dinucleotides are docking sites for regulatory proteins such as methyl-CpG binding protein-2 (MeCP2) and are generally thought to confer transcriptional repression, but this is not always the case [2]. The turtle *BDNF* coding exon IV and 3′ UTR also undergoes epigenetic modification during classical conditioning [25]; methylated CpG sites are significantly reduced after one session of conditioning. We are intrigued by the possibility that alternative splicing occurs in the *BDNF* coding exon at sites of methylation [26], [27]. While the promoter regions of the *BDNF* gene have received intense scrutiny for the presence of epigenetic alterations during activity and learning, the coding exon has been largely ignored. Since an alternatively spliced *tBDNF* transcript occurs in the coding region, the turtle *BDNF* gene presents a valuable opportunity to examine mechanisms of transcriptional regulation and alternative splicing. Another potential mechanism for transcriptional regulation of *tBDNF* splice variants during conditioning related to epigenetic marks and that have a high level of specificity is naturally occurring antisense transcripts. These non-coding RNAs are derived opposite to the sense strand. Recently, human and mouse *BDNF* antisense RNA has been shown to inhibit *BDNF* mRNA transcription by promoting alterations in chromatin structure [28]. Currently, however, evidence for antisense transcripts interacting with the rodent *Bdnf* gene is under debate [6].

### Possible Functions of the Truncated BDNF Protein

Expression of mature BDNF protein is recognized to be critically important in many forms of synaptic plasticity and learning [1]. Its alternative splicing and expression is regulated by epigenetic factors in models of aging [2], drug addiction [5] and disease states [29]. In the *in vitro* classical conditioning model used here, expression of full-length mature tBDNF protein is a required step in the signaling events that initiate postsynaptic AMPAR incorporation [12], [13], [17] and presynaptic structural modifications that underlie learning [18], [19]. The truncated tBDNF protein is expressed in resting and pseudoconditioned states and is suppressed when full-length mature tBDNF is expressed during conditioning. Simply increasing the level of neuronal activity by application of glutamate or KCl fails to induce the conversion from truncated tBDNF to full-length tBDNF protein expression, indicating that the paired stimuli specifically induce signaling cascades that activate this process. It is intriguing that truncated tBDNF is not re-expressed after extinction training that consists of the same randomly unpaired stimulus trials as during pseudoconditioning when it is highly expressed. This likely reflects extinction training as a form of new learning in which an association is made in the absence of reinforcement [20] and suggests that repression of truncated tBDNF is specifically related to the learning process. It is unknown whether truncated tBDNF actively inhibits the expression of full-length mature tBDNF and vice versa either at the mRNA or protein level. It is intriguing to consider the possibility that truncated tBDNF may be suppressive and full-length tBDNF permissive for the learning process. Further studies are required to determine the ubiquity of truncated BDNF splice variants across species and the mechanisms of regulation and function of this newly recognized protein.

## Supporting Information

File S1 Figure S1. The cDNA sequence of *tBDNF* exons I, II, and III is shown as a partial sequence here for *T. scripta elegans*. The complete sequence for *Chrysemys picta bellii* can be obtained in the NCBI GenBank database (accession number AHGY01342430.1). The sequence for the common coding exon IV is shown complete. A partial sequence for *tBDNF2a* exon IV is shown to illustrate the deletion and is highlighted in yellow. The start and stop codons are shown in bold and underlined. Polyadenylation signals (PAS) are underlined. Sites for PCR and RLM-RACE primers are indicated: blue, inner and outer primers for exons 1–3 (Table S2); pink, 5′ RACE (Table S1); orange, 3′ RACE (Table S1). Table S1. Primers used for *tBDNF* 3′ and 5′ RACE analysis. Table S2. Primers used for RT-PCR of *tBDNF* transcripts.
(DOC)
