# Supplementary material for: Identification of a Functionally Distinct Truncated BDNF mRNA Splice Variant and Protein in Trachemys scripta elegans
Source: PLoS One. 2013 Jun 25;8(6):e67141. doi: 10.1371/journal.pone.0067141 (PMC3692439; doi:10.1371/journal.pone.0067141)
Supplement: File S1 — Figure S1. The cDNA sequence of tBDNF exons I, II, and III is shown as a partial sequence here for T. scripta elegans. The complete sequence for Chrysemys picta bellii can be obtained in the NCBI GenBank database (accession number AHGY01342430.1). The sequence for the common coding exon IV is shown complete. A partial sequence for tBDNF2a exon IV is shown to illustrate the deletion and is highlighted in yellow. The start and stop codons are shown in bold and underlined. Polyadenylation signals (PAS) are underlined. Sites for PCR and RLM-RACE primers are indicated: blue, inner and outer primers for exons 1–3 (Table S2); pink, 5′ RACE (Table S1); orange, 3′ RACE (Table S1). Table S1. Primers used for tBDNF 3′ and 5′ RACE analysis. Table S2. Primers used for RT-PCR of tBDNF transcripts. (DOC) [file pone.0067141.s001.doc]

**Identification of a Functionally Distinct Truncated BDNF mRNA Splice Variant and Protein in *Trachemys scripta elegans***

Ganesh Ambigapathy, Zhaoqing Zheng, Wei Li and Joyce Keifer

**SUPPORTING INFORMATION**

(Figure S1, Tables S1 and S2)


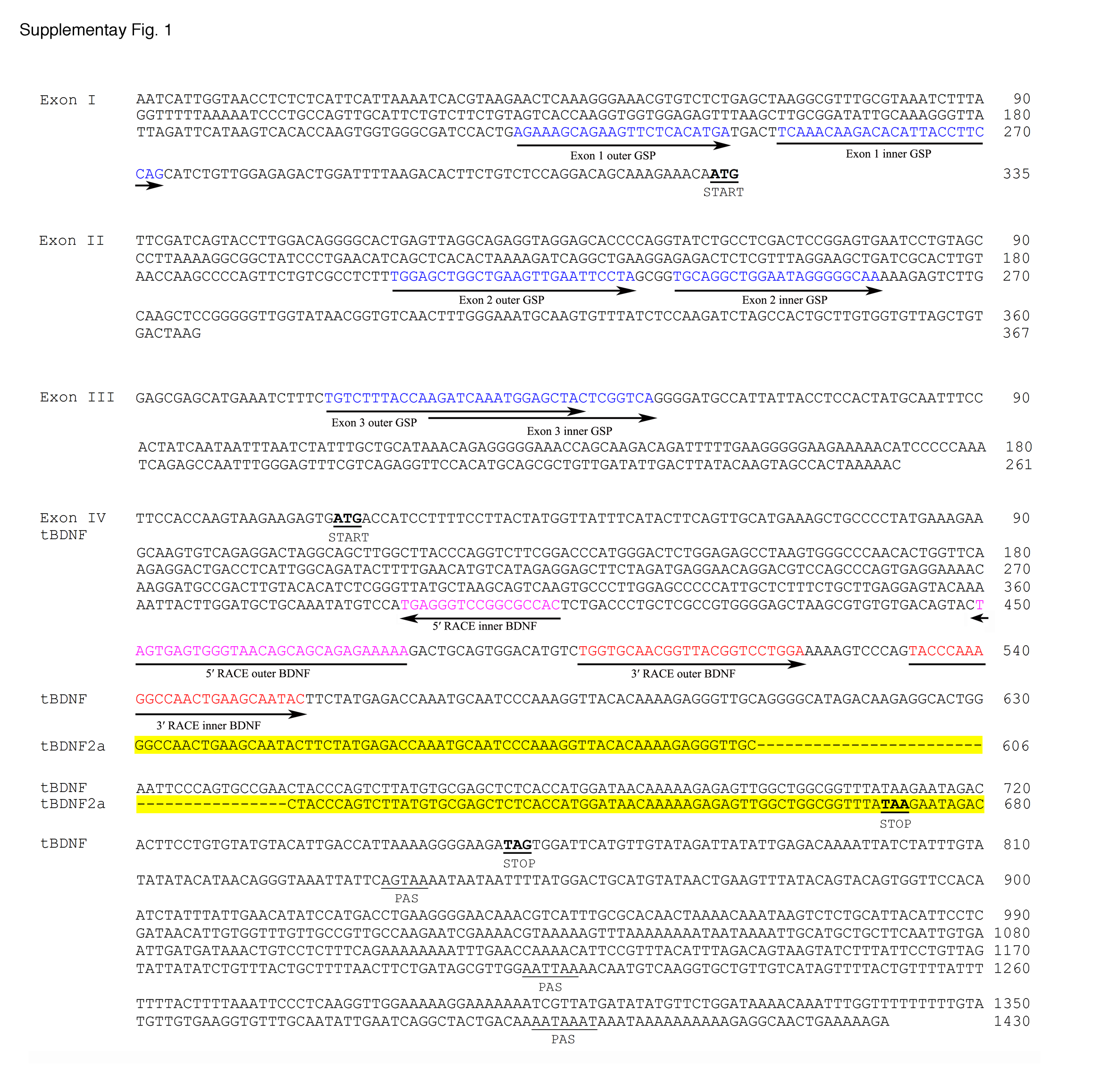


**Supplementary Fig. 1.** The cDNA sequence of *tBDNF* exons I, II, and III is shown as a partial sequence here for *T. scripta elegans*. The complete sequence for *Chrysemys picta bellii* can be obtained in the NCBI GenBank database (accession number AHGY01342430.1). The sequence for the common coding exon IV is shown complete. A partial sequence for *tBDNF2a* exon IV is shown to illustrate the deletion and is highlighted in yellow. The start and stop codons are shown in bold and underlined. Polyadenylation signals (PAS) are underlined. Sites for PCR and RLM-RACE primers are indicated: blue, inner and outer primers for exons 1-3 (Table S2); pink, 5’ RACE (Table S1); orange, 3’ RACE (Table S1).

**Table S1.** Primers used for *tBDNF* 3’ and 5’ RACE analysis.

| Primer | Orientation | Sequence |
| --- | --- | --- |
| BDNF | For | AGAAGAGTGATGACCATCCTTTTCCTTACTATGGTTA |
| BDNF | Rev | CTATCTTCCCCTTTTAATGGTCAATGTACATACACA |
| 3′ RACE outer BDNF | For | TGGTGCAACGGTTACGGTCCTGGA |
| 3′ RACE inner BDNF | For | TACCCAAAGGCCAACTGAAGCAATAC |
| 5′ RACE outer BDNF | Rev | TTTTTCTCTGCTGCTGTTACCCACTCACTA |
| 5′ RACE inner BDNF | Rev | GTGGCGCCGGACCCTCA |
| 3′ outer primer (Ambion) | Rev | GCGAGCACAGAATTAATACGACT |
| 3′ inner primer (Ambion) | Rev | CGCGGATCCGAATTAATACGACTCACTATAGG |
| 5′ outer primer (Ambion) | For | GCTGATGGCGATGAATGAACACTG |
| 5′ inner primer (Ambion) | For | CGCGGATCCGAACACTGCGTTTGCTGGCTTTGATG |

**Table S2.** Primers used for RT-PCR of *tBDNF* transcripts.

| Primer | Orientation | Sequence |
| --- | --- | --- |
| Exon 1 outer primer | For | AGAAAGCAGAAGTTCTCACATGA |
| Exon 1 inner primer | For | TCAAACAAGACACATTACCTTCCAG |
| Exon 2 outer primer | For | TGGAGCTGGCTGAAGTTGAATTCCTA |
| Exon 2 inner primer | For | TGCAGGCTGGAATAGGGGGCAA |
| Exon 3 outer primer | For | TGTCTTTACCAAGATCAAATGGAGCTAC |
| Exon 3 inner primer | For | AGATCAAATGGAGCTACTCGGTCA |
| 3′ outer primer (Ambion) | Rev | GCGAGCACAGAATTAATACGACT |
| 3′ inner primer (Ambion) | Rev | CGCGGATCCGAATTAATACGACTCACTATAGG |
